# Advances in the Synthesis and Application of Magnetic Ferrite Nanoparticles for Cancer Therapy

**DOI:** 10.3390/pharmaceutics14050937

**Published:** 2022-04-26

**Authors:** Seipati Rosemary Mokhosi, Wendy Mdlalose, Amos Nhlapo, Moganavelli Singh

**Affiliations:** 1 Nano-Gene and Drug Delivery Group, Discipline of Biochemistry, University of KwaZulu-Natal, Private Bag X54001, Durban 4000, South Africa; mokhosis@ukzn.ac.za; 2 Discipline of Physics, School of Chemistry and Physics, University of KwaZulu-Natal, Private Bag X54001, Durban 4000, South Africa; mdlalosew@ukzn.ac.za; 3 Department of Medical Physics, Sefako Makgatho Health Sciences University, P.O. Box 146, Pretoria 0204, South Africa; amos.nhlapo@smu.ac.za

**Keywords:** ferrites, cancer therapy, magnetic nanoparticles, biocompatibility, functionalisation

## Abstract

Cancer is among the leading causes of mortality globally, with nearly 10 million deaths in 2020. The emergence of nanotechnology has revolutionised treatment strategies in medicine, with rigorous research focusing on designing multi-functional nanoparticles (NPs) that are biocompatible, non-toxic, and target-specific. Iron-oxide-based NPs have been successfully employed in theranostics as imaging agents and drug delivery vehicles for anti-cancer treatment. Substituted iron-oxides (MFe_2_O_4_) have emerged as potential nanocarriers due to their unique and attractive properties such as size and magnetic tunability, ease of synthesis, and manipulatable properties. Current research explores their potential use in hyperthermia and as drug delivery vehicles for cancer therapy. Significantly, there are considerations in applying iron-oxide-based NPs for enhanced biocompatibility, biodegradability, colloidal stability, lowered toxicity, and more efficient and targeted delivery. This review covers iron-oxide-based NPs in cancer therapy, focusing on recent research advances in the use of ferrites. Methods for the synthesis of cubic spinel ferrites and the requirements for their considerations as potential nanocarriers in cancer therapy are discussed. The review highlights surface modifications, where functionalisation with specific biomolecules can deliver better efficiency. Finally, the challenges and solutions for the use of ferrites in cancer therapy are summarised.

## 1. Introduction

According to the World Health Organisation (WHO), cancer is among the leading causes of death worldwide, accounting for nearly 10 million deaths in 2020 [1]. This number is predicted to increase to about 12 million by 2030 [1,2]. Lung cancer accounted for as much as 1.80 million deaths in 2020, followed by colon and rectum (≈935,000 deaths), liver (≈830,000 deaths), stomach (≈769,000 deaths), and breast (≈685,000 deaths). The ability to diagnose early and provide treatment timeously may lead to a greater probability of survival and less morbidity [3]. The progress in nanotechnology has led to significant research developments within the medical and pharmaceutical industries. The application of nanomaterials is showing great potential in cancer therapy [4,5,6].

The evolution of systems designed for cancer diagnosis and treatment has enabled their application in magnetic resonance imaging (MRI), gene therapy, immunotherapy, and computed tomography [7,8,9]. Radiotherapy, chemotherapy, and surgery are still the choice for effective cancer treatments [10,11]. These conventional methods often fail for many reasons, ranging from complications that may develop post-administration to the adaptive nature of cancer cells. Surgery is invasive and can result in adverse side effects [2]. Furthermore, the exposure of the malignant tumours to chemical agents presents selective pressure that allows these cancerous cells to adapt, survive, and grow, resulting in their resistance to treatment [12]. Viral vectors have been the favoured gene delivery vehicles, owing to their high transfection efficiency and their naturally transducing nature [13,14]. Unfortunately, associated immunogenic responses to expressed viral proteins, possible integration into the host genome, challenges experienced in production, and toxicity have limited their use [13,15]. Hence, there exists a need for the design of more robust, efficient, and target-specific non-viral systems for gene delivery and cancer therapy.

**Figure 1:**
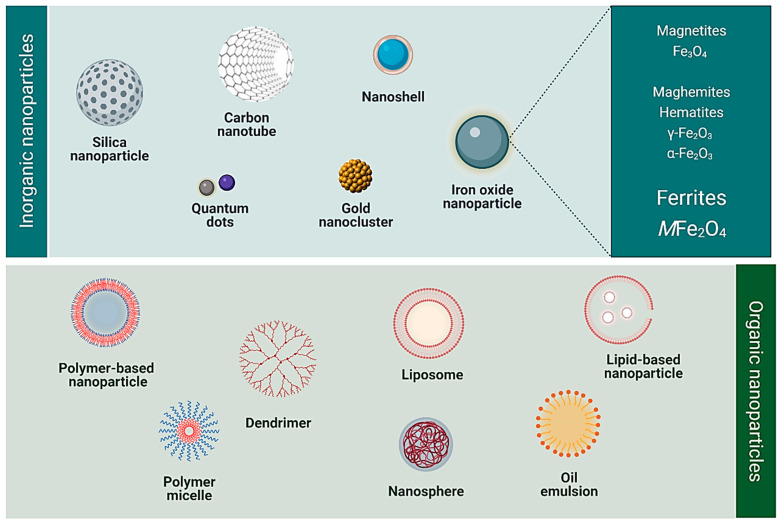
Various classes of organic and inorganic nanoparticles commonly used in nanomedicine (Created with BioRender.com, accessed on 4 January 2022).

Nanoparticles (NPs) can offer several advantages over other delivery systems, such as their facile synthesis, ease of functionalisation [7], and ability to be tailored for the desired application (for example, therapy and diagnostics) [9]. Furthermore, they can encapsulate hydrophobic molecules, such as anticancer drugs, and ensure increased solubility, biocompatibility, and specificity. Several NPs have emerged over the years for use in biomedical applications. These include organic linear polymers, hyperbranched polymers, dendrimers, liposomes, micelles, and inorganic NPs. Some inorganic NPs have been reported in preclinical and clinical studies to treat, diagnose, and detect certain diseases. These include gold, silica, carbon-based, and iron oxide-based magnetic NPs [16,17,18,19,20]. The commonly used inorganic and organic NPs in nanomedicine are illustrated in Figure 1.

Magnetic NPs comprise (i) metal NPs, such as Fe and Co; (ii) alloys including Au/Fe; and (iii) iron oxide-based NPs, viz., magnetite (Fe_3_O_4_), maghemite (γ-Fe_2_O_3_), and ferrites (e.g., CoFe_2_O_4_, MnFe_2_O_4_,and ZnFe_2_O_4_) [19,21,22]. Heavy-metal-containing NPs often present a challenge in their elimination from the body. However, several iron-oxide NPs have been approved for clinical use with minimal observed cytotoxicities [19,21]. Cobalt ferrite NPs coated with polymers such as polyvinyl alcohol (PVA), polyvinylpyrrolidone (PVP), and polyethylene glycol (PEG) have shown negligible cytotoxicity at concentrations up to 150 µg/mL [23]. An in vitro study reported that MgFe_2_O_4_ NPs coated with chitosan, polyethylene glycol (PEG), and polyvinyl alcohol (PVA) successfully delivered doxorubicin to colorectal and breast adenocarcinoma cells with minimal toxicity at concentrations of up to 100 µg/mL [24]. Sustained and targeted delivery of the anticancer drug 5-fluorouracil (5-FU) by chitosan-coated Mg_0.5_Co_0.5_Fe_2_O_4_ NPs to cancer cells rather than healthy cells was also reported [25].

ZnFe_2_O_4_ NPs have been preferred in some applications due to their biocompatibility and low toxicity at concentrations below 125 µg/mL [26]. The observed minimal toxicity is attributed mainly to the elemental composition of the NPs, making them more biocompatible and degradable. Furthermore, ideal magnetic NPs must demonstrate good magnetic properties such as high saturation magnetisation (MS) and low coercivity (HC), with uniform nano-sizes less than 100 nm [27]. The key points in the use of iron-oxide NPs for cancer therapy are summarised in the subsequent sections.

## 2. Iron Oxide Nanoparticles in Cancer Diagnostics and Therapy

Iron oxide NPs that possess characteristically large surface areas, small particle sizes, and superparamagnetism have been cited in applications geared towards diagnosis and targeted drug delivery [28,29,30]. When an external magnetic field is applied to superparamagnetic iron oxide NPs (SPIONs), the domains are aligned to the field. Once removed, the magnetisation of these NPs returns to zero. Another type of magnetic NP that has been explored in nanomedicine is ferromagnetic NPs. However, these NPs are permanently magnetised; hence, they retain the magnetisation even in the absence of the external field. When the external magnetic field is applied in both superparamagnetic and ferromagnetic NPs, their magnetic moments turn to flip in the direction of the applied field. This flipping of the magnetic moments generates heat, which forms the basis of tumour ablation therapy through hyperthermia [31,32].

Traditionally, magnetite (Fe_3_O_4_), hematite (α-Fe_2_O_3_), and maghemite (γ-Fe_2_O_3_) have been the most widely used. Magnetite NPs are the most predominant and present interesting properties due to their two valence states, Fe^2+^ and Fe^3+^ [11]. They possess ferromagnetic properties and exhibit a reverse cubic spinel structure. Their structure comprises tetrahedral (A) sites, octahedral (B) sites, and mixed tetrahedral/octahedral layers with iron ions Fe^2+^ and Fe^3+^. Half of the Fe^3+^ ions occupy A sites surrounded by four oxygen atoms, while the mixture of Fe^2+^/Fe^3+^ ions occupy site B and are surrounded by six oxygen atoms [32].

Polymer-based magnetic microspheres encapsulating magnetite NPs were first confirmed for magnetic drug delivery and targeting by Senyei et al. in 1978 [33]. Several studies have reported the successful synthesis of polymer-coated (chitosan, PEG, polyethyleneimine (PEI), dextran) magnetite NPs that demonstrated desirable properties for use as clinical MRI contrast agents [34,35]. Dextran coating was reported to improve the physical properties of SPIONs for use as MRI contrast agents and in hyperthermia therapy [36]. In another study, magnetite NPs modified with PEI and galactose were investigated for siRNA-targeted delivery in hepatocellular carcinoma, with promising results. Maghemite shares a similar spinel structure to magnetite; however, maghemite has a vacancy of divalent iron ions. The unit cell is slightly smaller than the magnetite cell due to the formation of cationic vacancies and the smaller size of Fe^3+^ ions than Fe^2+^ ions [37]. Citrate-coated maghemite NPs were investigated in vitro for combined magnetic hyperthermia and photodynamic therapy to treat selected cancers, including glioblastoma [38].

In one of the earliest clinical trials, polystyrene-coated iron oxide NPs were used to enhance MRI of the gastrointestinal tract (GIT). Table 1 represents a summarised report of iron oxide NPs in clinical trials that have been approved by the Food and Drug Association (FDA) since 1996 [18,39,40]. Typical examples of coating agents include dextran/carboxydextran in Ferumoxtran, Ferumoxide and Ferucarbotran; PEG in Feruglose; and aminosilane in Nanotherm™. GastroMARK^®^ utilises siloxane-coated iron oxide NPs for MRI of the GIT [41]. Ferumoxytol is a polyglucose-sorbitol-carboxymethyl-ether-coated γ-Fe_2_O_3_ that was developed and approved in 2000 as an MRI contrast agent for many cancers. The nature of the coating and the hydrodynamic size often determines the fate of NPs within a biological system. This includes the interactions of the coated iron oxide NPs with their cellular environment, uptake, accumulation, circulation, and clearance from the body [42]. 

**Figure 2:**
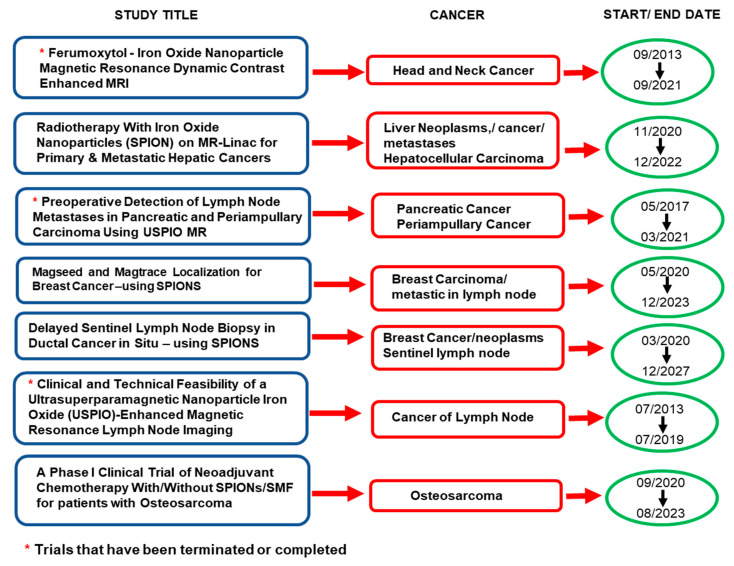
Highlights of the iron oxide nanoparticles used in clinical trails for cancer that have been completed or terminated from 2019 onwards, and those that are still continuing or recruiting (adapted from [43]).

Furthermore, Figure 2 highlights the studies using iron oxides for cancer that have advanced to clinical trials, with some already terminated or completed since 2019 [43].

**Figure 3:**
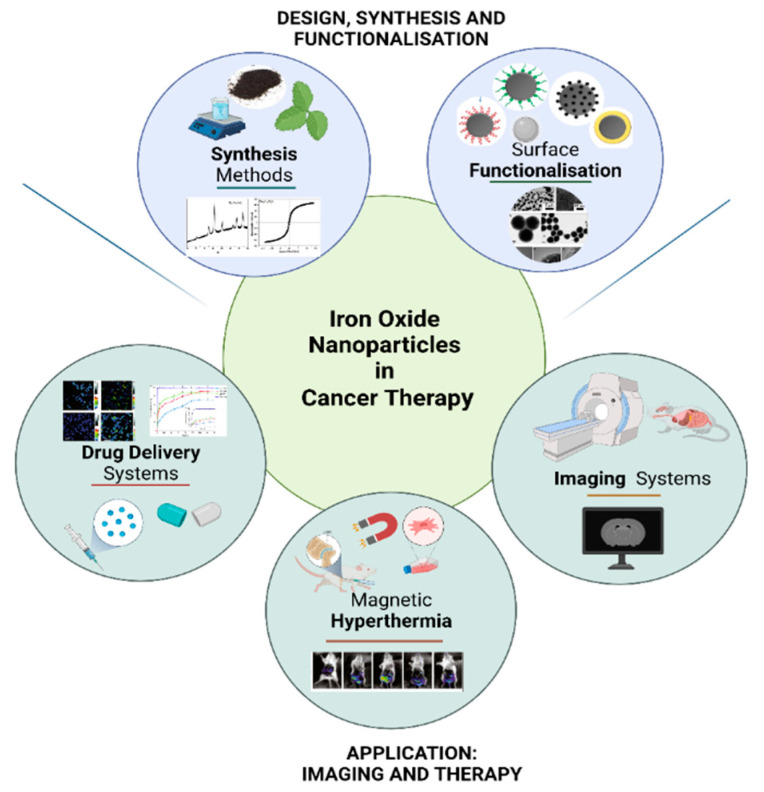
The application of iron oxide nanoparticles in cancer diagnostics and therapy (Created with BioRender.com, accessed on 12 March 2022).

With this advancement of iron oxide NPs to the clinical trial stage, it is evident that surface functionalisation, often with either surfactants or polymers, is a crucial prerequisite step when intended for medical applications such as MRI, hyperthermia, or drug delivery [22,44]. Figure 3 outlines the applications of iron oxide NPs in cancer diagnostics and therapy.

### 2.1. Magnetic Hyperthermia

In hyperthermia therapy, an alternating magnetic field (AMF) is applied to the NPs, resulting in elevated temperatures between 42 and 45 °C [45,46]. The heat is generated due to the magnetic hysteresis loss, Néel relaxation, and Brown relaxation. Hysteresis losses are observed in iron oxide NPs that have multiple magnetic domains. The resulting continuous alignment and differences in energy released can lead to heat generation [47]. On the other hand, in Néel relaxation, there are rapidly occurring changes in the direction of magnetic moments relative to the crystal lattice. This relaxation is delayed by the energy of anisotropy that tends to orientate the magnetic domain in a given direction relative to the crystal lattice [48].

In contrast, the Brownian relaxation results from the physical rotation of particles within a medium in which they are placed. The delay, in this case, is due to the stickiness that tends to counter particles’ movement in the medium [48]. The heat generation is influenced by parameters such as size, shape, and the magnetic properties of the NPs. At sizes of <20 nm, the heat release mechanism is by Neel relaxation, while larger NPs use the Brown relaxation mechanism [49]. At these temperatures, apoptosis is induced, as tumour cells are sensitive to temperatures above 41 °C due to their higher metabolic rates [50]. This method has been used complementary to the existing radiation and chemotherapy in cancer therapy [44,50,51]. As previously mentioned, the most widely used iron oxide NPs for magnetic hyperthermia are magnetite and maghemite NPs, owing to properties such as biocompatibility, non-toxicity, and excellent magnetisation [51].

Superparamagnetism in magnetic NPs has resulted in more heat generation than ferromagnetic NPs, owing to higher hysteresis losses from the single-domain structure [52]. Other important factors include the frequency and the square of the amplitude of the external AMF. It has been proposed that for safe application in patients, the amplitude should not be more than 5 × 109 Ams^−1^ [53]. With this targeted approach, the heating of cancer cells can be attained without damaging surrounding normal tissue, thus increasing the effectiveness and safety of hyperthermia. In a recent report, hyperthermia therapy was employed through utilising cobalt ferrite NPs where 90% doxorubicin delivery was achieved in 6 h at 44 °C [54]. Nanotherm^®^ is an FDA-approved formulation comprising iron oxide NPs coated with aminosilanes (Table 1) [55] and is indicated for hyperthermia treatment of the malignant glioblastoma multiforme [56,57]. The administered treatment consists of water-dispersed NPs with a diameter of 15 nm. Cancer cells subjected to the Nanotherm^®^ treatment have been found to exhibit greater sensitivity when complemented with radiotherapy or chemotherapy [56].

In contrast to magnetic hyperthermia, another useful strategy, referred to as nano-magnetomechanical activation (NMMA), which involves the low frequency (<1 KHz) activation of magnetic NPs in a non-heating alternating magnetic field, has been reported [58]. This approach can be employed to facilitate the release of therapeutic molecules from the functionalised magnetic NP carrier or to change the bound biomolecule’s chemical properties. This technique can produce site-specific tissue regeneration or lead to the destruction of malignant cells. Although this process has the advantages of being regulated, non-invasive, selective, and relatively safe, it needs the MNPs to undergo rotational oscillations [59], and the force used could stimulate tumour growth due to collateral impact on the surrounding normal tissue [60].

### 2.2. Targeted Drug Delivery

Since the coining of the term “magic bullet” by Paul Elrich in 1906 [61], significant research milestones have been reached in designing and developing targeted nanotherapeutics geared for enhanced and site-specific delivery [9,62]. An essential advantage of using iron-oxide-based NPs is the ease of preparation and functionalisation for tailored specificity [63]. Furthermore, introducing an external alternating magnetic-field-induced oscillation delivery facilitates magnetic targeting, with drug leakage or exposure of healthy cells to the drug until it reaches the target site [9]. The brief introduction of the external magnetic field circumvents challenges such as non-specificity in distribution and delivery, toxicity in healthy cells, and overall diminished therapeutic efficacies. Once the NP reaches the site, controlled drug release mechanisms often utilise parameters such as pH, light, thermal stimuli, and redox stimuli [9,64].

**Figure 4:**
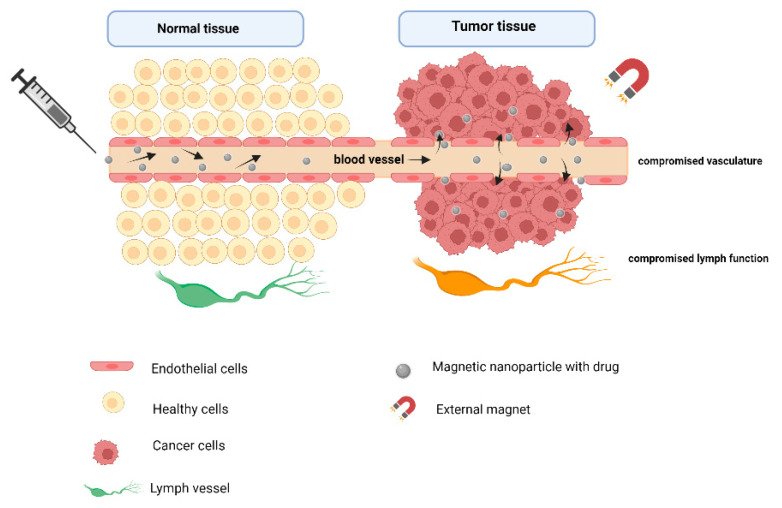
Illustration of the enhanced permeability and retention effect in cancer cells allows for passive targeting (Created with BioRender.com, accessed on 28 March 2022).

Owing to the nano-sizes of NPs, there is a tendency for passive accumulation in tumour tissues due to enhanced permeability and retention (EPR) [56]. This passive targeting exploits the compromised vasculature and the micro-tumour environment with different pH values and temperature and poor lymphatic drainage [65]. Figure 4 illustrates the changed vasculature in cancer cells that potentiate the EPR effect. 

Solid tumours, however, present with other challenges, such as tumour heterogeneity and matrix barriers, e.g., fibrosis collagen. In active targeting, NPs can be functionalised or tailored for specific ligand receptor recognition or antigen–antibody interactions [66,67]. The overexpression of certain receptors triggers receptor-mediated transcytosis found only in cancer cells, allowing for internalisation of the NP carrying the therapeutic [68]. Polymer-coated iron oxide NPs allow drug for encapsulation within the matrix, which can be trigger-released upon reaching a tumour site [69]. A significant number of reports have shown functionalised magnetites being suitable carriers of anticancer drugs such as doxorubicin, 5-FU, morin, and ciprofloxacin [70,71,72].

Recently, ferrites have emerged as suitable biocompatible NPs in drug delivery for anticancer treatment, with increased studies on ferrites for targeted drug delivery. A finding showed that manganese ferrite NPs coated with chitosan and PEG exhibited high encapsulation efficiency of methotrexate [73], while chitosan-functionalised Mg_0.5_Co_0.5_Fe_2_O_4_ NPs enhanced 5-FU delivery in MCF-7 cells in vitro [25]. A pH-responsive drug release was activated in both studies under acidic conditions. A similar stimuli-responsive drug delivery using ZnFe_2_O_4_ and Ag_1−x_Zn_x_Fe_2_O_4_ NPs was observed under different pH conditions [74]. The Zn_x_Mg_(1−x)_ Fe_2_O_4_ NPs for drug delivery were also investigated, confirming their good drug loading capacity and drug release profiles [75]. Furthermore, Gd3+ ion-doped CoFe_2_O_4_ NPs studied for targeted drug delivery and contrast enhancement in MRI showed sustained drug release of 90.6 to 95% over 24 h at a pH of 7.4 [76]. Lime peel extract was also used to produce NiFe_2_O_4_ NPs that were investigated as antioxidant, anticancer, and antibiotic agents [77]. In another study, bismuth-doped Ni-ferrites (NiFe_2−x_Bi_x_O_4_) NPs were synthesised via the co-precipitation method and proposed as being useful in targeting magnetic carriers [78].

### 2.3. Imaging Systems

MRI is a vital visualisation tool in clinical diagnostics, such as in detecting tumours. The effectiveness of MRI is influenced by the magnetic resonance signal of the examined tissues by the contrast agents. There are two major types of MR contrast agents: positive (T1-weighted agents) and negative (T2/T2-weighted agents) contrast agents. Positive contrast agents can shorten the longitudinal relaxation time (T1) of protons and result in a bright image. Negative contrast agents shorten the transverse relaxation time (T2) of protons and tend to decay rapidly in the traverse direction, leading to a dark image [45,50]. T1 gadolinium (Gd)-based contrast agents have been the most employed in clinical settings for brighter images with better resolution [46,79]. However, these display disadvantages such as short lifespan, poor cellular uptake, and risk factors in patients with kidney and liver diseases. The free Gd ions cannot be effectively cleared post-administration, resulting in toxicity in natural settings [58].

Superparamagnetic iron-oxide NPs have been widely employed and reported to exhibit higher MRI signal contrast than the Gd-based ones due to the high saturation magnetisation [45]. Additionally, by nature of the Fe being a biomineral used by the body, they may be biodegradable and biocompatible in vivo compared to metal or metal alloy-based contrast agents [11,21]. When coated with PEG, PVA, dextran, or modified chitosan, these NPs have been proven to be superior due to low long-term toxicity and long shelf lives [11]. Ferrites such as CoFe_2_O_4_ are currently being explored as T2 MRI contrast agents; however, more research is necessary to determine their cytotoxicities over time before clinical trials [34,80]. Their potential as T1 MRI contrast agents is currently under investigation, where parameters such as size, shape, and surface coatings need to be considered. For example, these NPs should optimally be < 5 nm for an excellent T1 image resolution [19,80]. Additionally, the type of surface modification on the NP affects the relaxation time [34]. It has been observed that hydrophilic coatings, which stabilise water molecules around the NPs, have an adverse effect on T2 by lowering it [21]. CoFe_2_O_4_@MnFe_2_O_4_ NPs were reported to enhance targeted MRI and fluorescent labelling in MGC-803 cell lines and tumours [81].

## 3. Ferrites (MFe_2_O_4_)—Nanocarriers of Choice in Biomedicine

Spinel ferrites have been explored for application in electromagnetics, information storage, and sensor technology [82,83,84]. In recent years, they have been gaining immense interest in biomedicine due to their optical and magnetic properties and the possibility of tailoring them by changing the initial composition or cation substitution [23,82].

Ferrite NPs are soft magnetic materials with the structural formula of spinel-type ferrites written as (M^2+^ _1−λ_Fe^3+^ _λ_) [M^2+^ _λ_Fe^3+^ _2−λ_]O_4_. The parentheses and square brackets denote cation sites of tetrahedral (A) and octahedral (B) coordination [85]. They possess a single-phase cubic spinel structure with the simplistic formula: MFe_2_O_4_, where M = Mg, Mn, Ni, Cu, Zn, Co, or Fe [83]. Mixed ferrites comprise mixtures of both oxidation states existing at both sites, as seen in MnFe_2_O_4_, while CoFe_2_O_4_ forms either the inverse or mixed spinel structure, dependent on the synthesis conditions such as time [80,86]. Being cubic means more octahedral sites are available than tetrahedral sites, and both sites have magnetic moments aligned in antiparallel directions, resulting in higher magnetisation. The idea is to combine the unique properties conferred by the substituted molecule, usually a divalent cation metal such as Mn, Co, Ni, or Ca, and those possessed by the iron oxide NPs. Such ferrites ultimately exhibit an excellent magnetic nature, large surface area, small size, and biocompatibility required for biomedical applications [79]. Ferrites are highly versatile, with a nano-size dimension that allows for controlled surface manipulation to yield highly selective NPs. The structural and magnetic properties of spinel ferrites strongly depend on the magnetic moment, particle size and distribution, shape, and crystallinity, which are highly sensitive to the preparation method [80,82,87,88].

**Figure 5:**
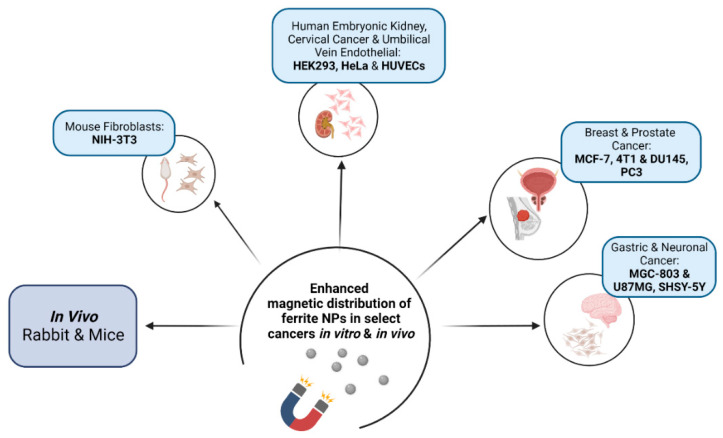
Cancer cell models and in vivo systems used in investigating the magnetic distribution of ferrite nanoparticles (Created with BioRender.com, accessed on 30 March 2022).

Ferrites were first investigated and reported for their biocompatibility in a study by Kuckelhouse et al. on cobalt-ferrite-based magnetic fluid and magnetoliposomes [89]. Following treatment with the NPs at selected doses and treatment time, no tissue damage was observed, and the capillaries and parenchymal cells were unaffected. Since then, there have been sporadic reports on ferrites in biomedicine. Synthesis of cobalt ferrite NPs for hyperthermic applications was reported by Baldi et al. [90]. Lin et al. also investigated the biocompatibility of Mn_0.5_Zn_0.5_Fe_2_O_4_ NPs for their potential in tumour hyperthermia, with toxicity studies indicating a good therapeutic effect on hepatocellular carcinoma cells in vitro and in vivo [91]. Figure 5 briefly highlights some of the cell models and in vivo systems where there has been enhanced distribution of ferrite NPs. 

Over the years, coating has become an essential requirement for improved biocompatibility of ferrite NPs. Table 2 reports the increased and robust research profiling of ferrites for cancer diagnostics and therapy that has been reported since 2015.

Even though the data has shown the feasibility of ferrites in cancer treatment and disease diagnosis, to date, none of the spinel ferrites have been approved or have advanced to the clinical trial stage [70]. The main challenge is the potential toxicities that may result from exposure. Some factors determine the structural, electrical, magnetic, and chemical properties of nano-ferrite particles and impact the biocompatibility and toxicity of the NPs [127]. The choice of synthesis methods often dictates particle size, crystallinity, composition, and site occupancy. Synthesis methods include sonochemical, mechanochemical, hydrothermal, co-precipitation, and sol–gel routes [23,86]. These are discussed in depth in Section 4.

## 4. Synthesis Methods of Ferrites

**Figure 6:**
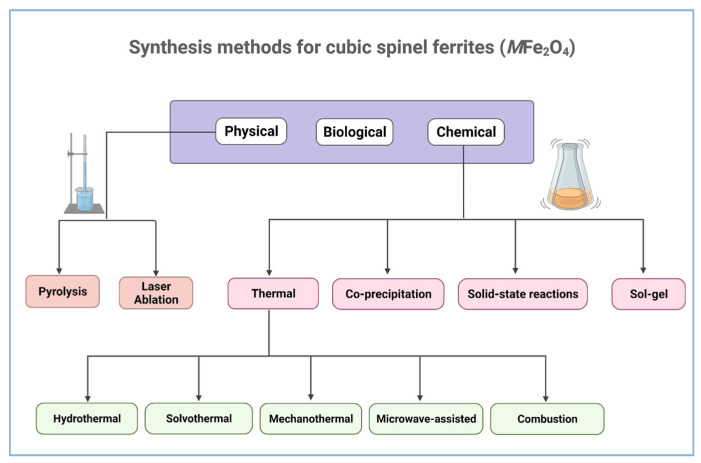
Synthesis methods for ferrite materials with cubic spinel structure (Created with BioRender.com, accessed on 2 January 2022).

In recent decades, developing a wide diversity of nanomaterial synthesis techniques has utilised the bottom-up and top-down approaches [128]. Ferrite NPs are synthesised using these methods to address the complex functionality required for human healthcare and medicine [86]. There are three main categories of synthesising magnetic NPs: biological, physical, and chemical methods, as shown in Figure 6 [129]. The choice of synthesis method plays a crucial role in the properties of the NPs depending on their intended applications. Uniformity of NPs is one of the important requirements as their structural, electrical, optical, and magnetic properties largely depend on their dimensions [130]. The methods listed in Figure 5 are the most common, reliable, and simpler synthesis methods for the effective fabrication of cubic spinel ferrites materials with desired properties [131,132].

### 4.1. Biological Methods

Biological methods used in green synthesis are thought to be easy and economical. They have received much attention as suitable alternatives to physical and chemical methods [133]. Extracts from different parts of the plants such as roots, leaves, stems, latex, fruit pericarp, fruit juice, and seeds have been used to synthesise ferrite NPs where they acted as stabilising and reducing agents [134]. Fungi, algae, and bacteria have been reported to facilitate the synthesis of ferrites NPs, providing a valuable and innovative biotechnological asset to produce highly stable NPs [135]. In particular, ferrite NPs of single-phase cubic spinel structure can be synthesised using a bacterial-mediated process in which metal-reducing bacteria transform pure Fe (III) oxyhydroxides plus soluble metal species or metal-substituted akaganeite into ferrite particles outside of the bacterial cells. Cobalt ferrite NPs with single-phase cubic spinel structures have also been successfully produced using yeast cells [133]. The biological synthesis techniques have gained attention because they are environmentally friendly, inexpensive, and require relatively low temperatures.

### 4.2. Physical Methods

Physical methods such as plasma, laser ablation, chemical vapour decomposition/evaporation decomposition, molecular beam epitaxy, and gamma radiation have also been reported to successfully produce ferrite NPs [136]. Among these methods, evaporation–condensation and laser ablation are the most important approaches due to fewer contamination possibilities. These techniques produce NPs with more uniformly distributed particle sizes than wet chemicals methods [135]. In addition, they can produce NPs with small grain sizes of about 100 nm, making them suitable for biomedical applications [137]. Sorescu et al. reported on the structural and magnetic properties of nanocrystalline NiZn and Zn ferrite thin films produced by laser ablation deposition [138]. Recently, Özçelik et al. studied the structural, magnetic, photocatalytic, and blood compatibility of Ni ferrites, where 30 nm and 50 nm particle sizes were obtained following the use of laser ablation in distilled water [139]. Pulsed laser ablation in liquid and sol–gel approaches were also employed to produce Co_0.5_Ni_0.5_Ga_0.01_Gd_0.01_Fe_1.98_O_4_/ZnFe_2_O_4_ spinel ferrite NPs, with sizes ranging from 55 to 80 nm [140].

### 4.3. Wet Chemistry Methods

Wet chemistry methods utilise chemical reactions in the liquid phase to synthesise NPs. These methods are unique and reliable and provide a high level of control and reproducibility for the fabrication of magnetic ferrite NPs. Each chemical synthesis technique is unique, offering various advantages and disadvantages. Low-temperature reactions are preferred for the synthesis of ferrite NPs. Most magnetic NPs used in biomedical applications have been synthesised using the co-precipitation method. Other methods include the glycol-thermal/solvothermal [141,142], hydrothermal [143,144], auto-combustion and co-precipitation [145,146], sol–gel [147], forced hydrolysis [148], CTAB-assisted and microwave-assisted hydrothermal methods, and the citrate precursor method [149,150]. These methods produce shape-controlled and un-agglomerated mono-dispersed NPs. The hydrothermal, solvothermal, and co-precipitation methods share a common feature. Both require stoichiometric amounts of metal chlorides or nitrates of high percentage purity as starting materials [151]. The different types of wet chemistry methods and their advantages are discussed below.

#### 4.3.1. Co-Precipitation

The co-precipitation technique is widely used to synthesise soft ferrites in biomedical applications. This method offers many advantages, such as low reaction temperature, high product yield, and the use of environmentally friendly solvents such as water. Furthermore, it improves cation distribution, leading to uniform and homogeneous particles with lower porosity and narrow size distribution [152]. Briefly, Fe^3+^ precursors in the form of chlorides or nitrates are mixed in water, along with a surfactant such as oleic acid, under rapid stirring and gentle heating. After adjusting the pH, a precipitate of the ferrite NPs is formed, which is washed to remove the chlorites or nitrates. The obtained precipitate is dried in a hot oven (80–100 °C) to burn the carbonaceous matter in order to leave a residue of the ferrite NPs [153], which is then ground into a powder. Pre-sintering and post-sintering are done at a suitable temperature to obtain the desired NPs [86]. Parameters such as pH, heating rate, heating atmosphere, and sintering temperature need to be tailored to their end application [154]. Ferrite NPs prepared by the co-precipitation method have also been reported to display good electromagnetic properties, thus making them useful in multi-layer chip industries [118]. Ni-doped barium ferrite sizes between 14 and 16 nm were reported using the co-precipitation method [155].

#### 4.3.2. Thermal Methods

Synthesis of magnetic NPs such as ferrites sometimes requires high temperatures to meet the high-quality standards [156]. The process starts with the production of metal chlorides or nitrates as precursors, followed by a reaction under high temperatures. The various thermal methods include hydrothermal, solvothermal, microwave, mechano-chemical, and combustion [157].

##### Hydrothermal and Solvothermal Methods

These methods are the most straightforward, effective, and inexpensive thermal approaches to synthesising ferrite NPs. Appropriate parameters such as temperature, pressure, and reaction time can result in excellent sample quality [158]. In both these methods, Fe^3+^ and divalent metal M^2+^ salts (in the form of chlorides or nitrates) are used as precursors. The salts are dissolved in water under rapid stirring, with a base gently added to form a precipitate. Following the pH regulation, the mixture is filtered to remove the chlorides or nitrate ions, then placed in an autoclave or pressure reactor and cooled to room temperature. For the glycol-thermal method, water is replaced with ethylene glycol. After heating, the cooled mixtures are washed, dried, and homogenised [159,160]. A significant advantage of the hydrothermal method is that it can be integrated with other processes such as the microwave, electrochemistry, ultrasound, and optical radiations to increase the ability to produce new materials [161]. With this technique, the particle growth and shape can be monitored by optimising the reaction time, temperature, reactant concentration, type of solvent, and precursors. The hydrothermal method was used to synthesise Ni_0.65_Zn_0.35_Cu_x_Fe_(2−2x/3)_O_4_ ferrites with sizes between 10 and 17 nm [155]. 

##### Microwave Method

The microwave synthesis technique is a relatively new method of synthesising ferrite NPs other than synthesis techniques [128]. The microwave-assisted chemical reaction has received wide attention in recent years [162,163,164,165]. This method has many advantages, such as faster heating, higher reaction rate, a higher degree of crystallisation, higher product yield, and smaller particle sizes with a narrower distribution [166]. The rapid structural formation under microwave could be associated with confined super-heating of the solutions [134]. This method provides a chance of synthesising ferrite NPs on a broader scale depending on the application [128,167]. The structural and magnetic properties of Co_1.0_Fe_2.0_O_4_, Ni_0.9_Fe_2.1_O_4_, Cu_1.1_Fe_1.9_O_4_, and Zn_1.1_Fe_1.9_O_4_ ferrites produced by the microwave-hydrothermal method showed the formation of a cubic spinel structure [157]. Furthermore, Mg_x_Cd_1−x_Nd_0.03_Fe_1.97_O_4_ ferrites were successfully synthesised by the microwave sintering technique with NP sizes between 39 and 40 nm [168].

##### Mechano-Chemical Method

This method is based on the processing of solids where mechanical and chemical reactions are coupled on a molecular scale [169]. It is characterised by repeated deformation, fracture, and welding of the mixture of chemicals and uses reactions of solid acids based on hydrated compounds, crystal hydrates, and basic and acidic salts. The chemical reaction in this technique is usually carried out at high temperatures, followed by lower temperatures during the ball milling [170]. The selection of suitable synthesis conditions such as stoichiometry and the chemical reaction route of starting materials and milling conditions is essential. Lazarević et al. successfully synthesised single-phase cubic spinel Zn and Ni ferrite NPs of 10 and 15 nm in size [171]. In comparison, Castrillón Arango et al. synthesised Ni_1−x_Co_x_Fe_2_O_4_ (0 ≤ x ≤1) crystalline ferrites with sizes from 16 to 32 nm using the mechano-chemical method [172].

##### The Combustion Method

This is an easy, simple, and economical method for synthesising ferrite NPs. It also requires reduced time and energy spent during the synthesis process. In this technique, stoichiometric compositions of high purity metal chlorides or nitrates are mixed under oxidising agents such as urea, glycine, and hydrazides [173]. The fuel glycerine is used as a reducing agent that drives the combustion [174]. The batches are placed in a glassy silica dish and homogenised to produce a slurry due to the nature of the metal nitrates. This is then heated, with the combustion reaction process taking about 20 min, although the actual time of ignition can be less than 10 s. During combustion, foams are produced, yielding a voluminous and fluffy product [175] that is often pure and homogeneous. The method of Jagadeesha et al. was used to synthesise Mn_0.5_Zn_0.5_Fe_2_O_4_, Mn_0.5_Zn_0.5_Fe_1.95_Sm_0.05_O_4_, and Mn_0.5_Zn_0.5_Fe_1.9_Gd_0.05_Sm_0.05_O_4_ NPs with sizes of about 10 nm [176].

#### 4.3.3. The Sol–Gel Method

This multistep method involves hydrolysis, condensation, and polymerisation reactions of metal precursors, and consequently gel formation [177]. This method offers the advantage of good stoichiometric control, resulting in ultrafine high purity NPs with a narrow size distribution within a relatively short processing time at low temperatures. Sol–gel is also easy and cost-effective. Briefly, an aqueous solution of metal salts is co-precipitated by a metal hydroxide or a base, then treated to form a colloidal sol or an inorganic or metallo-organic precursor, which can then be concentrated into a gel and subsequently fired to produce the fine-grained polycrystalline ferrite NPs [178]. Recently, pure spinel Ni ferrite NPs were prepared by the sol–gel method using polyacrylic acid (PAA) as a chelating agent. The size distribution, specific surface area, and crystallinity of ferrite nanoparticles were controlled by varying the molar ratios of PAA to total metal ions and calcination temperature [134]. Furthermore, MnYb_y_Fe_2−y_O_4_ ferrites with a single-phase cubic spinel structure from 24 to 80 nm in size were synthesised by the sol–gel method [131]. 

#### 4.3.4. Solid-State Method

This method has ease of operation, is cost-effective, and is suitable for the mass production of NPs. It primarily involves mixing the raw metal oxides or metal salts of high purity stoichiometrically with a few drops of methanol and then grinding them using a high-energy ball machine over time [179]. The most important benefit of this method is that samples can be synthesised without any solvent [180]. The synthesis of Co_1−x_Zn_x_Fe_2_O_4_ (x = 0 and 0.5) spinel ferrite NPs using different methods showed that the solid-state method readily produced particle sizes at the nanoscale [181].

## 5. Potential of Ferrites in Biomedical Applications

**Figure 7:**
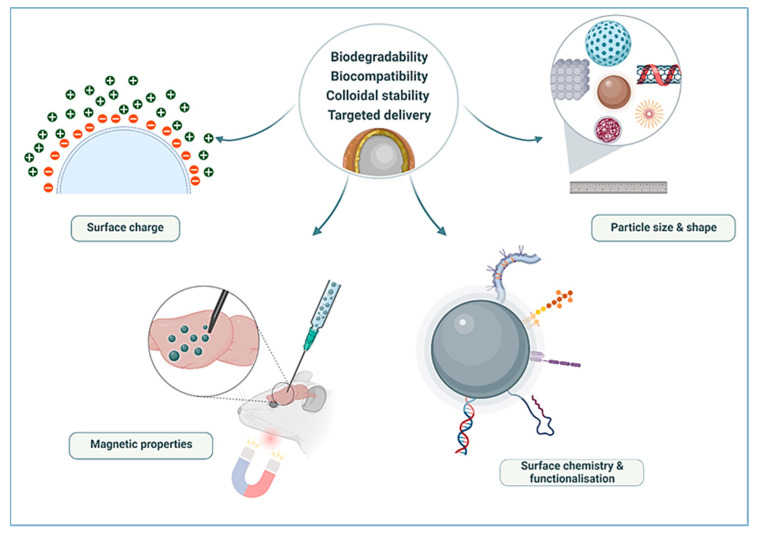
Parameters to be considered for the application of magnetic NPs in biomedical applications (Created with BioRender.com, accessed on 13 January 2022).

The recent surge in research on ferrites in drug delivery has been encouraging as they present a great potential for broader use in biomedicine. To be considered for biomedical applications, nanocarriers should demonstrate biodegradability, biocompatibility, and stability in vivo, as highlighted in Figure 7. 

Physical parameters that influence the applicability of these NPs include magnetic properties, size, drug/gene binding capacity, and physiological parameters such as internal trafficking to target vascular supply and body weight. Optimisation of these factors can result in a desired and precise therapy [182]. Several other parameters such as shape, stoichiometry, and surface structure were also reported [183] to influence the physical and chemical properties of magnetic NPs. Much of these properties are dependent on the synthesis method and chemical composition [152,184]. These parameters and their influence on the use of ferrite NPs in biomedicine are further addressed below.

### 5.1. Particle Size and Shape

The size of magnetic NPs is a major physical property used to tailor magnetic properties and their surface area. Many researchers have explored the controlled size synthesis of iron oxide NPs and found that sizes below 100 nm display superparamagnetism [156,185,186,187]. Superparamagnetic NPs become magnetic in the presence of an external magnet but revert to a non-magnetic state when the external magnet is removed. This behaviour prevents the activity of the particles when there is no applied field, leading to less particle aggregation [37], which is advantageous for in vivo applications [188]. The boiling point of the solvent and reaction time during synthesis are the significant factors in determining the NP size, as evidenced by the variation in sizes of superparamagnetic NPs reported [189,190,191]. Furthermore, the importance of the hydrodynamic sizes of NPs has been observed to influence the injectability, biodistribution, and clearance of hyperthermia or MRI agents [192]. However, the synthesis of magnetic NPs of favourable sizes has been a scientific and technological challenge to date. 

The magnetic properties of the NPs also depend to a great extent on their shape. The change in shape can lead to different crystal surfaces and atomic arrangements, which affect the overall properties of the NP [67]. These physical properties can be controlled by the NP’s chosen synthesis method or design. A theoretical study suggested that the NP shape affects a particle’s circulation half-life in the blood [193]. This was supported in a study that showed that filamentous polymer micelles have long-circulating lifetimes (>one week) after administration, while spherical counterparts lasted for about 2 to 3 days [194]. These results were mainly attributed to the tendency of the particles to align with blood flow. Further reports have suggested that spherical and cubic magnetic metal oxide NPs possessed different magnetic properties [195]. Spherical NPs have exhibited superparamagnetic behaviour and reduced aggregation in many studies [196,197]. One of the main challenges in biomedical applications of magnetic NPs is their toxicity. A recent report suggested that rod-shaped magnetic NPs are less toxic than spherical shaped NPs [198], further highlighting the vital role that size and shape play in the properties of the NPs.

Physical synthesis methods such as gas-phase deposition and electron beam lithography are elaborate procedures that suffer from the inability to control the size and shape of particles in the nanometre size range [199]. However, the wet chemical syntheses routes are simpler, more manageable, and more efficient, with appreciable control over size, composition, and sometimes even the shape of the NPs [200]. Several protocols have been reported that can control the size and shape of desired NPs [130,201]. Due to the dissimilarity in the properties of the NPs resulting from different synthesis routes, the development of protocols for desired morphology, size, and shape is still under consideration. Wu et al. suggested that the size and dynamics of synthesised NPs can be controlled by altering the concentrations of iron oxide precursor to base, solvents, and surfactants; nature of surfactants; initial concentration of reactants; and reaction temperature and time [22].

### 5.2. Surface Chemistry and Functionalisation

NP surfaces are tailored to improve biocompatibility, solubility, and stability; reduce aggregation; improve size distribution; and reduce toxicity to healthy cells and tissues [130,199]. Additionally, the interactions of NPs with the immune system, plasma proteins, and extracellular matrices is a critical consideration influenced by both charge and hydrophobicity [80]. Hence, the surface chemistry of the NPs is a crucial factor that affects their biocompatibility and biodegradability. Several studies have reported on coated ferrite NPs with good biocompatibility in drug delivery and hyperthermia [22,199,202,203]. The coating can be achieved using organic biopolymers or inorganic layers [204,205,206]. The nature of the coating and the hydrodynamic size can affect the fate of the NPS in a biological system, such as cellular uptake and accumulation, circulation, and clearance from the body. Moreover, appropriate surface functionalisation is necessary for conjugating biomolecules to NPs for desired applications [44,46,207].

#### 5.2.1. Organic Polymers

Biopolymers used in NP surface functionalisation commonly comprise natural and synthetic polymers. Magnetic NPs are often coated with organic polymers such as chitosan, dextran, PVA, PEG, and PVP [44,130,208,209]. One of the most notable roles of polymers is to prevent oxidation, which leads to effective absorption by body tissues, thus providing better stability during therapy. Dextran is a natural polymer with a neutral, branched polysaccharide comprising glucose subunits, rendering it highly biocompatible. Dextran and its derivatives (carboxydextran, carboxymethyl dextran) have been the earliest and most frequently used polymers in many clinically approved iron oxide-based NPs for MRI and hyperthermia therapy [45,69]. 

Ramnandan et al. compared the different coatings (chitosan, PEG, and PVA) on MgFe_2_O_4_ NPs to deliver doxorubicin in vitro [24], showing that chitosan produced the most favourable results. The same polymers were investigated on Mg_0.5_Co_0.5_Fe_2_O_4_ NPs for their cytotoxicity in selected cells lines [108,109]. Chitosan is a linear polysaccharide made from chitin and contains important reactive functional groups such as amines. Not only is chitosan known to improve biocompatibility and biodegradability, but it also provides a way to conjugate therapeutics, imaging agents, and targeting ligands [25,29]. PVA and PEG are synthetic organic polymers with attributes that include enhanced particle monodispersity that inhibits particle coagulation [24,29]. PVA is water-soluble and demonstrates emulsifying and adhesive properties, leading to reduced particle aggregation [203]. PEG is also well reported for its role in ensuring long circulation in biological systems [12,35]. The resulting steric hindrance and stabilisation by the polymer allow NPs to escape from the reticuloendothelial system (RES) [210,211]. 

#### 5.2.2. Inorganic Compounds

Silica, carbon, metals, and oxides are the most widely explored inorganic coating compounds for iron-oxide NPs that have been used in biomedical applications [22]. Notably, there are fewer inorganic materials available for magnetic NP surface coatings. Gold (Au) and silica (SiO_2_) have traditionally been the most employed due to their biocompatibility and unique properties [212]. Silica is very stable and presents stability over a wide pH range. It is inert and improves the dispersion of NPs by creating a layer that buffers the dipolar attraction between the NPs. The resultant minimised aggregation of NPs leads to better stability [213]. It is imperative to select appropriate coating methods in silica-coating as this will indirectly impact the NP’s therapeutic efficacy. Silica coating also allows for conjugation of other biomolecules, ligands, fluorophores, dyes, and quantum dots [212]. The most commonly employed methods are the Stöber method, microemulsion, and aerosol pyrolysis. During the Stöber process, silica is formed by the hydrolysis and condensation of a sol–gel precursor, resulting in iron oxide@SiO2 NPs. A homogeneous silica layer must be produced without core-free silica particles as these may lead to a reduced efficacy dose of NPs in MRI and hyperthermia treatment. By modulating the silica shell thickness, the efficiency of the NPs as contrast agents can be accomplished [22]. Several studies have investigated the use of Au-coated iron oxide hybrid NPs for the unique properties conferred by both materials [32,213]. On their own, AuNPs exhibit positive attributes such as facile synthesis, good stability, and unique optical properties. When used to coat iron oxide NPs, they can prevent corrosion that may result at low pH. Moreover, they demonstrate localised surface plasmon resonance, making them ideal agents for imaging or photothermal ablation in cancer therapy [32]. The gold shell further prevents oxidation of the iron oxide core and provides an excellent surface functionalisation chemistry [214]. Similar to the silica layer, shell thickness and geometry need to be carefully tuned as this can impact on the magnetic properties of the NPs, which may result in their lowered proficiency as MRI contrast agents [213].

A grave challenge often encountered when developing a drug delivery system for cancer therapy is the inability to reach therapeutic levels of the drug at disease sites. One of the reasons is the nonspecific uptake of NPs by healthy organs. Furthermore, NP interactions with opsonins and attachment to plasma proteins result in a corona formation [215]. Opsonisation is primarily influenced by NP hydrophobicity and charge. Hence an understanding of the surface chemistry and the impact of the coating is crucial. Hydrophobic and charged NPs have demonstrated shorter circulation times due to opsonisation, leading to RES recognition and rapid elimination from circulation [211]. Additionally, the hydrophobic groups on the surface of NPs can lead to the agglomeration of NPs and be quickly removed by the RES. Thus, the surface properties of the NPs, such as charge, are vital to limiting NP–host interactions [215]. Several studies on the physical and magnetic properties of the naked and coated magnetic NPs have been performed, seeking to develop coating methods that enhance the properties of the NPs and ensuring consistently better cellular uptake and efficient therapy.

## 6. Conclusions

Cancer theranostics to date remains an urgent and vital research niche, given the rising mortality rates caused by various cancers globally. There has been exponential progress in developing nanotherapeutics to address challenges related to conventional therapies. The main challenges for applying NPs for cancer therapy include limited cellular uptake, which is also dependent on size and shape. To limit elimination by the RES before reaching the target site, these parameters need to be carefully modulated. As magnetic NPs are inherently predisposed to aggregation and agglomeration, which may directly impact the stability and toxicity of NPs, their modifications to circumvent these issues need to be optimised. While these modifications, such as in polymer coating, remain critical, they should not compromise the therapeutic efficacy of the delivery system. Ideally, magnetic NPs should exhibit superparamagnetic behaviour, with high saturation magnetisation and low coercivity. Ferrites have emerged as an exciting subclass of iron-oxide NPs in nanomedicine. This is attributed to their unique and advantageous ultra-structural and magnetic properties. Furthermore, as iron is an essential mineral in the body, it is expected to undergo the natural degradation processes in the body, reducing any adverse cytotoxicity. The various synthesis routes for spinel ferrites have been highlighted, as these often play a role in the ultra-structural characteristics of NPs. Incorporating biopolymers such as PEG, PVA, and chitosan has become standard practice for designing NPs geared for nanomedicine as they generally contribute to the overall biocompatibility of the NPs. While current studies have paved the way for potentiating ferrites in cancer theranostics, more effort is required to optimise size, shape, surface properties, and drug loading capacities, which are paramount to the success of magnetic NPs as therapeutic delivery vehicles.

## Figures and Tables

**Table 1:** Iron oxide NPs in clinical trials for cancer imaging and therapy (adapted from [18,39,40]).

Trade/Generic Name/Clinical Trial ID	Nanocomposite Material	Application (Cancer Type)
Abdoscan^®^/Ferristene/OMP (Nycomed)	Polystyrene-coated iron oxide NPs	MRI imaging: gastrointestinal tract
Combidex^®^ (USA), Sinerem^®^ (EU), Ferumoxtran-10/AMI-277 (Guerbet/AMAG Pharmaceuticals Inc)	Iron oxide coated with dextran (T10)	MRI imaging: prostate, breast, bladder, genitourinary cancers, and lymph node metastases
Feraheme^®^ (USA), Rienso^®^ (EU)/Ferumoxytol (AMAG Pharmaceutical Inc.)	Polyglucose-sorbitol-carboxymethyl-ether-coated iron oxide (γ-Fe_2_O_3_)	Imaging: rectal, oesophageal, bone, colorectal, prostate, bladder, kidney, lymph node, head and neck, breast, non-small cell lung, and pancreatic cancers; osteonecrosis, soft tissue sarcoma, chondrosarcoma, glioblastoma; melanoma
Feridex I.V. (USA), Endorem™ (EU), AMI-25/ferumoxides (AMAG Pharmaceuticals)	Iron oxide coated with dextran (T10)	MRI—liver/spleen imaging
Lumirem^®^ (USA), GastroMARK^®^ (EU), AMI- 121 (AMAG Pharmaceuticals Inc/Guerbet)	Siloxane-coated iron oxide NPs	MRI Imaging: gastrointestinal tract
Resovist^®^ (USA, Japan, EU) Cliavist^®^ (France), Ferucarbotran/ SHU555A (Bayer Schering Pharma)	Carboxydextran-coated iron oxide (γ-Fe_2_O_3_)	MRI imaging: liver/spleen tumours
Nanotherm™ (Magforce Nanotech AG)	Aminosilane-coated iron oxide NPs	Thermal ablation, hyperthermia local ablation in glioblastoma.
MagProbe^TM^ (University of New Mexico)	Magnetic iron oxide NPs	Leukaemia
Magnablate I (University College London)	Iron NPs	Prostate cancer
NC100150/Clarisan/Feruglose/PEG-fero (Nycomed)	Carbohydrate-polyethylene-glycol-coated ultra-superparamagnetic iron oxide NPs	MRI imaging: tumour microvasculature
Sienna+^®^ (Endomagnetics Ltd.)	Carboxydextran-coated iron oxide NPs	Breast and rectal cancer
NCT01895829 NTC03179449 NTC04369560	Polyglucose sorbitol carboxy methyl ether coated SPIONs	MRI detection for the spread of head and neck cancer MRI detection of inflammation (macrophage) in childhood brain neoplasm MRI detection for urinary bladder neoplasms
NCT01749280 NCT04316091	USPIONs	MRI to predict the growth of abdominal aortic aneurysms Neoadjuvant chemotherapy+ SPIONs/spinning magnetic field; evaluate tolerability, safety, and efficacy of the treatment: osteosarcoma
Ferumoxytol USPIO-MRI	Enhanced MRI	Enhanced MRI in imaging lymph nodes in patients with locally advanced rectal cancer: head and neck cancer
Ferumoxytol MIONs	Ferumoxytol	Pilot feasibility study of MIONs MR dynamic contrast-enhanced MRI for primary and nodal tumour imaging in locally advanced head and neck squamous cell carcinomas

**Table 2:** Literature reports on synthesis methods and surface functionalisation of ferrite NPs in cancer diagnostics and therapy (2015–2020).

Ferrites	Synthesis Method	Surface Functionalisation	Application	Reference
Iron oxide	Coprecipitation; sono-chemical	PEG	Potential bioapplication	[83]
Cobalt core @ manganese shell	Thermal decomposition	PEG	MRI and fluorescent labeling in vitro and in vivo	[81]
Cobalt and nickel	Solvothermal	Amine	Drug delivery	[92]
Cobalt and zinc–cobalt	Co-precipitation	Sodium citrate	Cytotoxicity in NIH-3T3 cell line	[84]
Cobalt	Solvothermal	L-Arginine	Drug delivery	[93]
Cobalt	Solvothermal	Leucine	Drug delivery	[54]
Cobalt	Sol–gel autocombustion	PEG	Potential bioapplication	[94]
Cobalt	Solvothermal	Folic acid	Hyperthermia	[95]
Cobalt	Microwave-assisted	Hydroxyapatite	Hyperthermia	[96]
Cobalt	Co-precipitation	-	Hyperthermia	[97]
Cobalt	Co-precipitation	PEG	Potential bioapplication	[98]
Cobalt	Solvothermal	-	Potential hyperthermia	[99]
Cobalt	Co-precipitation	Xantham gum, poly-methacrylic acid (PMAA)	Drug delivery	[100]
Cobalt	Co-precipitation	Xantham gum	Drug delivery	[101]
Cobalt	Hydrothermal	-	Potential bioapplication	[102]
Cobalt, copper, manganese, and nickel		Chitosan	Anti-cancer activity in MCF-7 cell line	[103]
Cobalt–manganese	Combustion	PEG	Potential bioapplication	[88]
Cobalt–zinc	Co-precipitation	DMSA	MRI in human prostate cancer cells	[104]
Copper–cobalt	Co-precipitation	-	Potential bioapplication	[105]
Copper–cobalt	Co-precipitation	-	Potential bioapplication	[106]
Magnesium	Combustion	Silica	Potential bioapplication	[107]
Magnesium–cobalt	Glycol-thermal	Chitosan, PEG, PVA	Cytotoxicities in HEK293 and HeLa cell lines	[108]
Magnesium–cobalt	Glycol-thermal	Chitosan, PEG, PVA	Cytotoxicities in HeLa cell lines	[109]
Magnesium–cobalt	Glycol-thermal	Chitosan	5-FU delivery fin HEK293, HeLa, and MCF-7 cell lines	[25]
Magnesium–manganese	Sol–gel, thermal decomposition	-	Potential bioapplication	[110]
Manganese–cobalt	Glycol-thermal	Chitosan	Potential bioapplication	[111]
Manganese	Sonochemical	Graphene oxide	Drug delivery	[112]
Manganese	Co-precipitation	Chitosan, PEG	Drug delivery	[73]
Cobalt and copper-doped manganese	Co-precipitation	Carboxymethyl cellulose	MRI, drug delivery	[113]
Manganese, gallium	Sol–gel	-	Potential hyperthermia	[114]
Manganese	Sol–gel self-combustion	-	Cancer therapy for murine breast cancer cell line (4T1)	[115]
Manganese–nickel	Microwave combustion	-	Potential bioapplication	[116]
Manganese, zinc, nickel, and cobalt	Hydrothermal	-	Potential bioapplication	[117]
Nickel	Co-precipitation, gas-phase condensation	-	Potential bioapplication	[118]
Nickel	Co-precipitation	Chitosan, PEG	Thermo-therapeutic applications	[119]
Nickel	Green synthesis, hydrothermal	-	Anti-cancer in neuronal cells	[120]
Nickel, zinc, and nickel–zinc	Thermal decomposition	Starch	Potential bioapplication	[121]
Zinc	Polyol	-	In vitro hyperthermia	[122]
Zinc	Green synthesis	-	Potential bioapplication	[123]
Zinc–cobalt	Co-precipitation	Dextrin	MRI	[124]
Zinc–magnesium	Glycol-thermal	-	Potential bioapplication	[125]
Zinc–manganese	Glycol-thermal	-	Potential bioapplication	[126]

## Data Availability

Not applicable.
